# Marek’s disease virus protein kinase US3 inhibits DNA-sensing antiviral innate immunity via abrogating activation of NF-κB

**DOI:** 10.1128/spectrum.02347-24

**Published:** 2025-03-05

**Authors:** Kai Li, Rui Liu, Yongzhen Liu, Li Gao, Changjun Liu, Yanping Zhang, Xiaole Qi, Hongyu Cui, Suyan Wang, Yuntong Chen, Yulu Duan, Yulong Gao, Xiaomei Wang

**Affiliations:** 1 Avian Immunosuppressive Diseases Division, State Key Laboratory for Animal Disease Control and Prevention, Harbin Veterinary Research Institute, Chinese Academy of Agricultural Sciences, Harbin, China; 2 Institute of Urban Agriculture, Chinese Academy of Agricultural Sciences, Chengdu, China; 3 Chengdu National Agricultural Science and Technology Center, Chengdu, China; 4 Jiangsu Co-innovation Center for Prevention and Control of Important Animal Infectious Disease and Zoonoses, Yangzhou University 38043 , Yangzhou, China; Shandong First Medical University, Jinan, Shandong, China

**Keywords:** Marek’s disease virus, US3, innate immunity, DNA sensing, NF-κB

## Abstract

Marek’s disease virus (MDV) is an avian alphaherpesvirus associated with Marek’s disease, an immunosuppressive and lymphoproliferative disease in chickens. The DNA sensing pathway mediates innate immune defense against infection by many DNA-containing pathogens, while viruses have evolved multiple strategies to evade the host immune response to survive in host cells. This study found that ectopic expression of MDV protein kinase US3 inhibited beta interferon (IFN-β) and interleukin-6 (IL-6) production induced by interferon-stimulatory and viral DNA. US3 was further shown to abolish the nuclear factor κB (NF-κB) activation. The US3 kinase activity was indispensable for its inhibitory function, as the kinase-dead US3 mutant (US3K220A) did not inhibit NF-κB activation. Further studies showed that US3 interacted with the Rel homology domains of the NF-κB subunits p65 and p50, which phosphorylated these transcription factors and blocked their nuclear translocation. Finally, US3 deficiency promoted IFN-β and IL-6 production, resulting in reduced viral replication and lower MDV-specific lesion incidence during MDV infection in chickens. Altogether, these findings reveal a novel mechanism for MDV to evade host antiviral immunity.

**IMPORTANCE:**

Marek’s disease virus (MDV) is an oncogenic avian alphaherpesvirus that causes an economically important disease affecting the health and welfare of poultry worldwide. Whereas human herpesviruses have been shown to evolve various strategies to inhibit the DNA sensing signaling for the evasion of the host’s innate immunity, little is known regarding the mechanism for MDV to regulate this pathway. In this study, MDV US3 protein kinase was demonstrated to inhibit the activation of NF-κB in the DNA sensing pathway via binding to the Rel homology domains of the NF-κB subunits p65 and p50, which hyperphosphorylated these transcription factors and abolished their nuclear translocation. This is an important finding toward a better understanding of the functions of avian alphaherpesviruses encoded US3 protein kinase.

## INTRODUCTION

Marek’s disease virus (MDV), an avian herpesvirus, is the etiological agent of Marek’s disease (MD), which is associated with the rapid induction of lymphomas and different kinds of nonneoplastic syndromes in chickens (1, 2). MDV is classified as a member of the alphaherpesvirinae subfamily, which also includes animal herpesviruses such as pseudorabies virus and bovine herpesvirus type 1, as well as human herpesviruses such as herpes simplex virus type 1 and 2 (HSV-1 and -2) and varicella-zoster virus. Despite the use of vaccines, MDV infection still circulates in poultry flocks. Susceptible chickens become infected with MDV through the respiratory route via the inhalation of infectious dander shed by infected chickens (3). After early cytolytic replication in macrophages and B cells, the virus causes latent infection of T lymphocytes, which are subsequently transformed, resulting in deadly lymphomas in the visceral organs (4). Despite many advances in understanding MDV pathogenesis, how MDV infection influences the innate and adaptive immune response remains to be investigated.

Innate immunity is the first line of host defense against infections by inducing type I interferon (IFN-I) and a wide range of antiviral effectors (5, 6). Viral nucleic acids represent important pathogen-associated molecular patterns that are recognized by host pattern recognition receptors (PRRs) to activate the IFN-I pathway, which ultimately induces the expression of multiple IFN-stimulated genes (ISGs) and instigates innate antiviral responses (7, 8). Recent studies have identified several cytosolic DNA sensors that recognize microbial pathogen DNA (9, 10). Among these DNA sensors, the cyclic GMP-AMP synthase (cGAS) is the predominant cytosolic DNA sensor recognizing a variety of DNA ligands in different cell types (11). Upon sensing viral DNA, cGAS catalyzes the second messenger cyclic GMP-AMP (cGAMP) synthesis, which interacts with and activates the stimulator of interferon genes (STING). Activated STING then recruits TANK-binding kinase 1 (TBK1) to phosphorylate the transcription factor interferon regulatory factor 3 (IRF3) and nuclear factor κB (NF-κB). Consequently, IRF3 and NF-κB translocate into the nucleus to induce the production of IFN-I and several inflammatory cytokines (12, 13).

Although the cytosolic DNA-sensing pathway is activated during viral infection, the herpesviruses have developed multiple mechanisms to evade host antiviral innate immunity (14–16). The Kaposi’s sarcoma-associated herpesvirus vIRF1 protein and human cytomegalovirus protein UL82 inhibit STING activation by preventing its binding to TBK1 or impairing its subcellular trafficking (17, 18). Recent studies have shown that HSV-1 can evade the DNA-sensing pathway at various levels, including recognizing through multiple DNA sensors and innate immune signaling through the IRF3 or NF-κB axis (19). The evasion of antiviral innate immunity is essential for viral persistent infection and replication in host cells.

Birds are an important reservoir of viruses causing human infections. Chicken PRRs differ from their mammalian counterparts in the absence of TLR9 and RIG-I (20). Comparatively, chicken, duck, and goose cGAS exhibited shortened N-termini with the least similarity of 22.4%, 17.4%, and 7.7% to human cGAS, respectively (21, 22). Chickens are IRF3 deficient, while the presence of functional IRF7 is considered to compensate for the IRF3 deficiency (20). NF-κB transcription factor is expressed in chickens and may be functionally similar to that in mammals (20). Like in mammals, the cGAS-STING axis plays a critical role in restricting DNA virus infection in avians (22). Nevertheless, how chicken DNA viruses, such as MDV, infectious laryngotracheitis virus, and fowl adenovirus evade cGAS-STING mediated antiviral signaling is still obscure.

MDV encodes a serine/threonine protein kinase US3 conserved in the alphaherpesvirus subfamily. US3 orthologs contain a kinase activity domain consisting of an ATP-binding domain and an active catalytic site, which is important for its kinase activity (23). Increasing evidence indicates that alphaherpesvirus US3 plays important roles in multiple processes during viral infection, including virion maturation, nuclear egress, apoptosis inhibition, cell-to-cell spread, and cytoskeletal rearrangements (24–28). HSV-1 US3 is also involved in viral immune evasion by inhibiting IFN-I production and downregulation of major histocompatibility complex class I surface expression (29–31). Although the functions of US3 have been extensively studied in other alphaherpesviruses, the biological functions of MDV US3 in the modulation of chicken innate immunity and its substrates have not been studied in detail.

In this study, we aimed to identify the cellular substrates of MDV US3 in the DNA sensing pathway and to investigate the role of MDV US3 in the evasion of host innate immunity. Our results show that MDV US3 interacts with and hyperphosphorylates the NF-κB subunits p65 and p50, which suppresses the nuclear translocation and activation of NF-κB, thereby leading to a blockade of IFN-I production. Our studies reveal a novel mechanism for MDV to evade host antiviral immunity.

## RESULTS

### MDV US3 inhibits IFN-β and IL-6 production induced by ISD, cGAS-STING, and DNA virus

**Fig 1:**
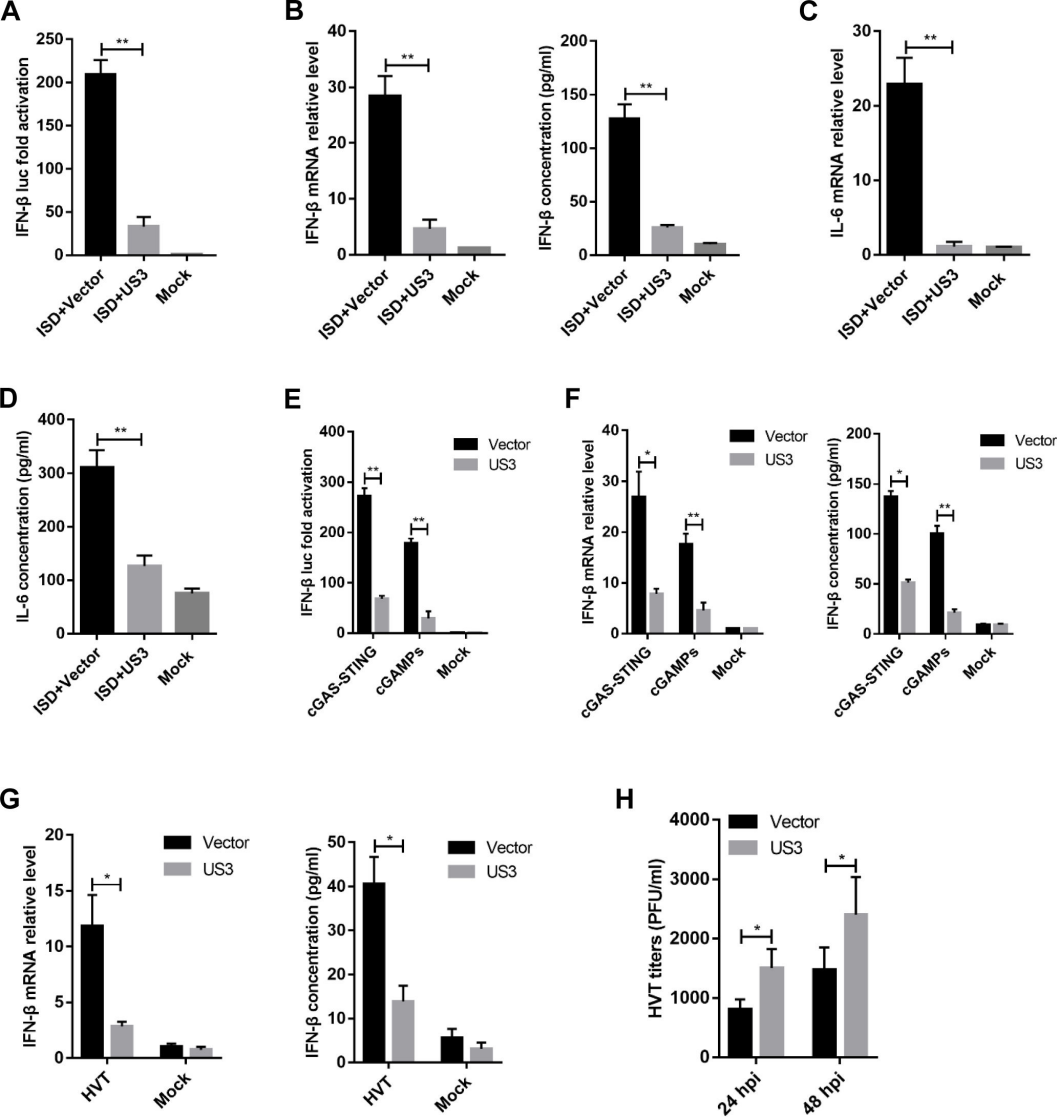
MDV US3 inhibits IFN-β and IL-6 production induced by ISD, cGAS-STING, and DNA virus. (**A**) IFN-β-Luc reporter was co-transfected with ISD and a US3 expression plasmid or empty vector into DF-1 cells; 24 h later, cells were harvested and subjected to a dual-luciferase reporter assay. (**B**) DF-1 cells were transfected with US3 expression plasmid or empty vector for 24 h and then transfected with ISD; *IFN-β* mRNA levels were measured by qPCR 8 h later, and *IFN-β* protein levels were measured by ELISA 24 h later. (**C, D**) DF-1 cells were transfected with US3 expression plasmid or empty vector for 24 h and then transfected with ISD; *IL-6* mRNA levels were measured by qPCR 12 h later, and *IL-6* protein levels were measured by ELISA 24 h later. (**E**) The cGAS and STING expression plasmids were co-transfected with US3 plasmid or an empty vector, and the IFN-β-luc reporter into DF-1 cells, and IFN-β promoter luciferase activity was measured 24 h later. (**F**) The cGAS and STING expression plasmids were co-transfected with US3 expression plasmid or empty vector into DF-1 cells, and *IFN-β* mRNA and protein levels were measured by qPCR and ELISA 24 h post-transfection. (**G**) DF-1 cells were transfected with US3 expression plasmid or empty vector and then left uninfected or infected with HVT (MOI = 0.1). *IFN-β* mRNA levels in these cells were measured by qPCR 12 h post-infection (hpi), and IFN-β protein was measured by ELISA 24 hpi. (**H**) DF-1 cells transfected with US3 plasmid or empty vector were infected with HVT (MOI = 0.01). At 24 hpi or 48 hpi, HVT viral titer was tested by plaque assays. The relative amount of *IFN-β* and *IL-6* mRNA was normalized to *actin* mRNA levels in each sample, and the fold differences between the treated samples and the mock controls were calculated. *P < 0.05, **P < 0.01.

DF-1, a chicken fibroblast cell line, is known to respond to foreign DNA such as interferon-stimulatory DNA (ISD) and poly(dA·dT) (32). To verify the role of MDV US3 in the regulation of IFN-β production, we transfected DF-1 cells with the exogenous DNA-sensing stimuli ISD and an US3-expressing plasmid or empty vector (EV), along with IFN-β promoter-luciferase reporter plasmids and then used dual-luciferase reporter (DLR) assays to detect IFN-β promoter activity. As shown in Fig. 1A, the ectopic expression of US3 markedly inhibited ISD-triggered IFN-β promoter activation. Furthermore, the mRNA and protein levels of IFN-β in cells transfected with these fragments were measured by real-time quantitative PCR (qPCR) and enzyme-linked immunosorbent assay (ELISA). IFN-β mRNA and protein levels were significantly increased in the EV-transfected cells in response to DNA stimuli, which was attenuated in US3-expressing DF-1 cells (Fig. 1B), suggesting that US3 inhibits the cytosolic DNA-induced IFN-β production. In addition, the production of IL-6 was also reduced by the ectopic expression of US3 in DF-1 cells (Fig. 1C and D).

To determine whether US3 could inhibit the cGAS-STING-mediated IFN-β production, the US3 expression plasmid was transfected into DF-1 along with cGAS and STING expression plasmids and the IFN-β promoter-reporter plasmids. As shown in Fig. 1E, the ectopic expression of US3 significantly inhibited cGAS-STING-mediated activation of the IFN-β promoter. Furthermore, IFN-β transcription and protein levels were significantly reduced in US3-expressing DF-1 cells (Fig. 1F). The results further showed that US3 inhibited IFN-β promoter activation and IFN-β production induced by cGAMP, the second messenger of the cGAS-STING signaling pathway (Fig. 1E and F). These results indicated that MDV US3 inhibits the cGAS-STING-mediated DNA sensing pathway. We then tested the effects of US3 ectopic expression on the production of IFN-β triggered by DNA virus infection. We infected US3-expressing DF-1 cells with herpesvirus of turkey (HVT) and found that US3 expression led to a diminished IFN-β response to this DNA virus compared to that observed in control cells (Fig. 1G). The infected HVT exhibited a higher replication titer in the US3-expressing cells (Fig. 1H). Altogether, these results indicated that the cGAS-STING DNA sensing signaling pathway was suppressed by MDV US3.

### MDV US3 inhibits activation of NF-κB

**Fig 2:**
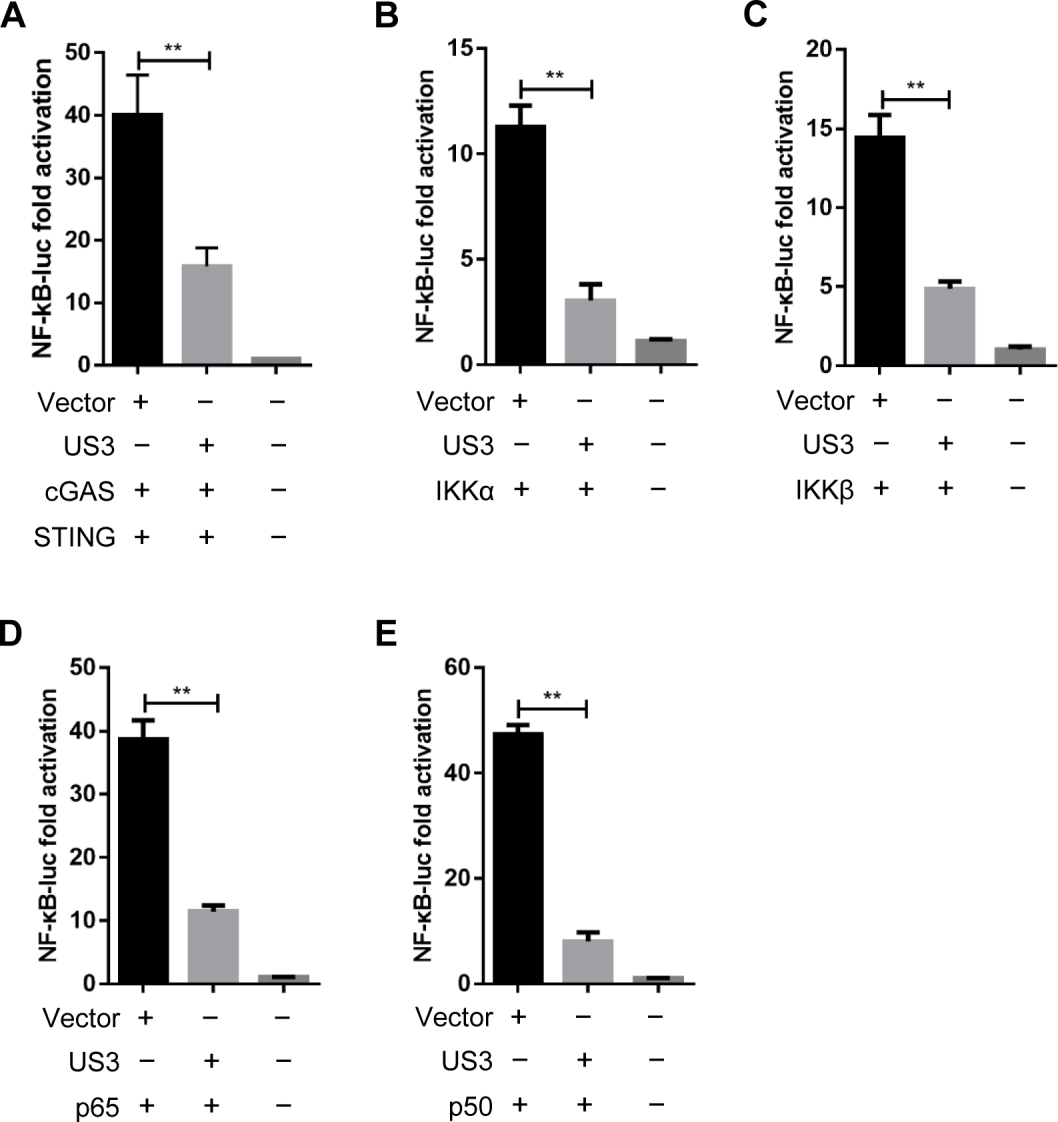
MDV US3 inhibits activation of NF-κB. (**A**) The cGAS and STING expression plasmids were co-transfected with the NF-κB-Luc reporter, as well as US3 expression plasmid or empty vector, into DF-1 cells; 24 h later, cells were subjected to the dual-luciferase reporter assay. (**B–E**) NF-κB-Luc reporter was transfected with plasmids expressing IKKα (**B**), IKKβ (**C**), p65 (**D**), or p50 (**E**) together with US3 plasmid or an empty vector. Dual-luciferase reporter assays were performed 24 h post-transfection. **P < 0.01.

The transcriptional activators NF-κB are essential for inducing IFN-I and other cytokines in chicken cells. To clarify the mechanism of IFN-β suppression by MDV US3, we analyzed the effects of US3 on NF-κB activation using a previously described DLR assay (32). The results showed that US3 inhibited NF-κB-dependent luciferase activity mediated by cGAS-STING (Fig. 2A), suggesting that US3 could inhibit the activation of NF-κB in the DNA sensing pathway. To further clarify the pathway components targeted by US3 during IFN-β activation, DF-1 cells were co-transfected with US3 expression plasmid, the NF-κB-Luc reporter plasmids, and expression plasmids encoding NF-κB signaling pathway components including IKKα, IKKβ, p65, and p50. All NF-κB adaptor proteins resulted in an approximate 10- to 40-folds induction of NF-κB-Luc reporter activity. However, NF-κB activation mediated by all NF-κB signaling pathway components was inhibited by US3 (Fig. 2B through E). These findings indicate that US3 probably suppresses the DNA sensing pathway by targeting all the NF-κB subunits.

### MDV US3 kinase activity is essential for inhibition of the DNA sensing signaling

**Fig 3:**
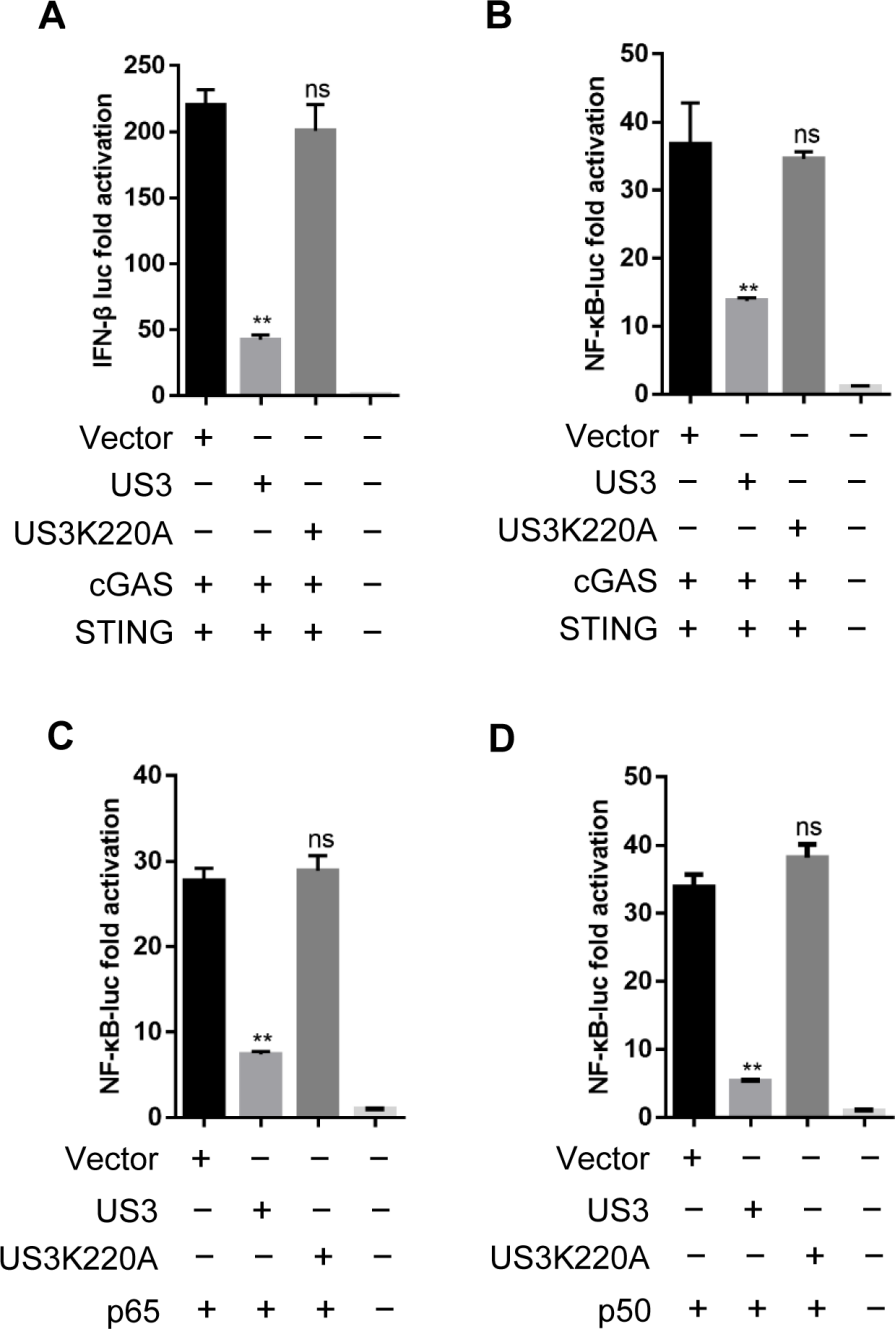
MDV US3 kinase activity is essential for inhibiting DNA sensing signaling. (**A, B**) The cGAS and STING expression plasmids were co-transfected with IFN-β-Luc (**A**) or NF-κB-Luc (**B**) reporters, as well as plasmid encoding US3 or a US3 kinase-dead mutant US3K220A or empty vector, into DF-1 cells; 24 h later, cells were subjected to the dual-luciferase reporter assay. (**C, D**) NF-κB-Luc reporter was transfected with plasmids expressing p65 (**C**) or p50 (**D**) together with US3, US3K220A expression plasmid or empty vector. Dual-luciferase reporter assays were performed 24 h post-transfection. ns, no significant difference, **P < 0.01.

To determine whether the kinase activity of US3 is required for inhibition of the DNA sensing signaling, we generated a kinase-dead US3 mutant US3K220A as previously described (33). Expression plasmids were co-transfected into DF-1 cells and examined for their ability to inhibit the cGAS-STING-mediated IFN-β and NF-κB reporter gene activities. The results showed that ectopic expression of wild-type US3 inhibited cGAS-STING-mediated activation of IFN-β and NF-κB reporters. However, ectopic expression of the US3K220A mutant failed to inhibit the IFN-β and NF-κB promoter activation (Fig. 3A and B). Furthermore, the US3K220A mutant had no significant effect on p65- and p50-induced NF-κB-Luc activity (Fig. 3C and D). These results suggested that the kinase activity of US3 is essential for inhibiting the DNA sensing signaling.

### MDV US3 interacts with p65 and p50

**Fig 4:**
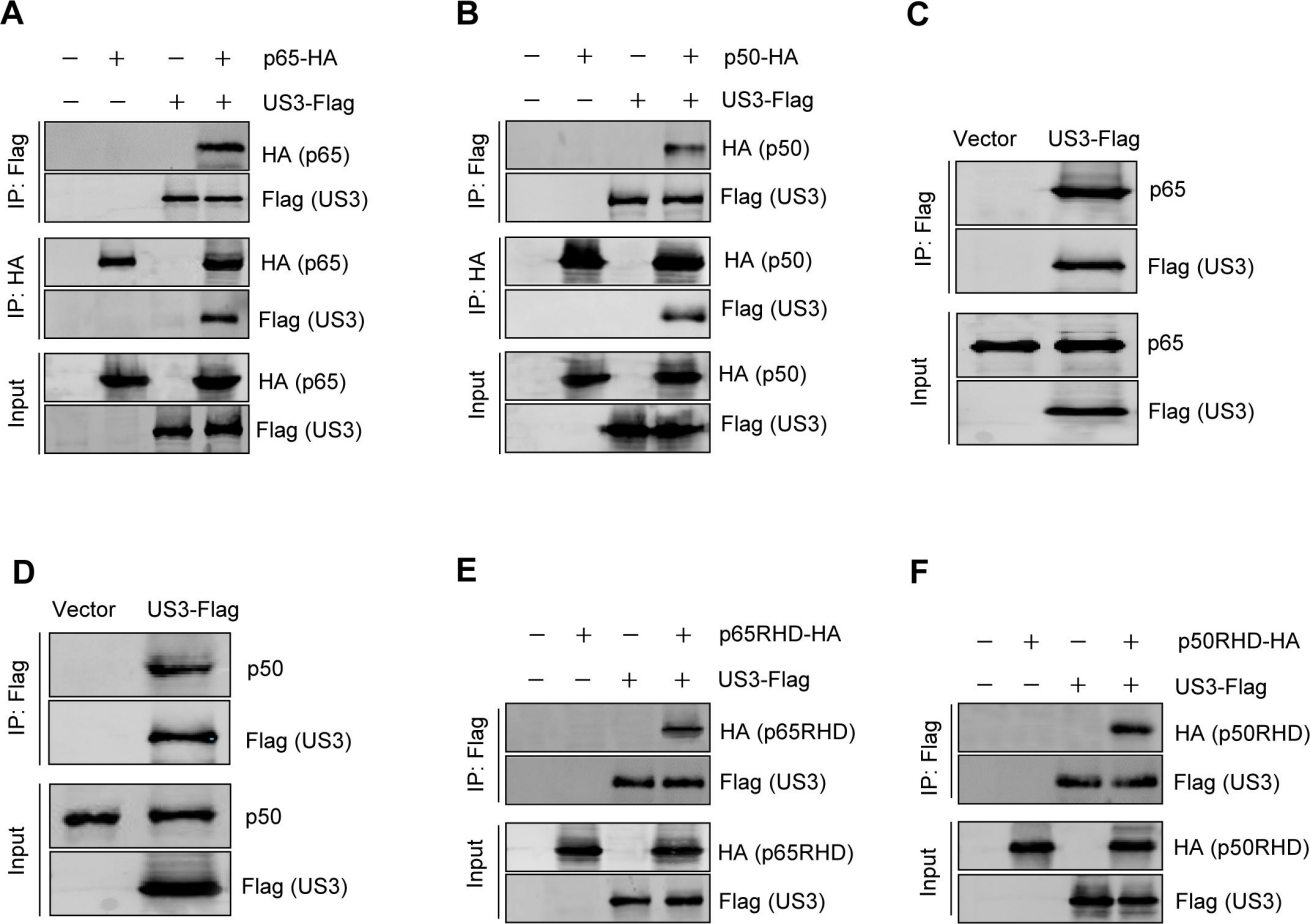
MDV US3 interacts with p65 and p50. (**A, B**) HEK293T cells were transfected with the indicated plasmids for 36 h before coimmunoprecipitation and immunoblot analyses with the indicated antibodies. (**C, D**) DF-1 cells were transfected with US3-Flag expression plasmid or an empty vector, and 36 h posttransfection, a coimmunoprecipitation assay was performed with an anti-Flag antibody. (**E, F**) HEK293T cells were co-transfected with US3-Flag and p65RHD-HA (**E**) or p50RHD-HA (**F**) plasmids. At 36 h post-transfection, cells were harvested, and coimmunoprecipitation assays were performed using anti-Flag antibodies.

To elucidate the molecular mechanisms through which US3 suppresses the DNA sensing pathway, we investigated the possibility of an interaction between US3 and the signaling pathway components. HEK293T cells were transfected with US3-Flag and p65-HA, or p50-HA, and a coimmunoprecipitation assay was performed with anti-HA and anti-Flag antibodies. We found that US3 was immunoprecipitated by p65 and p50; reciprocally, p65 and p50 were also immunoprecipitated by US3 (Fig. 4A and B). Furthermore, endogenous p65 and p50 were also immunoprecipitated by US3 (Fig. 4C and D). We also investigated the interaction of US3 with IKKα and IKKβ, and found no interaction of US3 with them (Fig. S1).

Both p65 and p50 contain a Rel homology domain (RHD) critical for protein dimerization, DNA binding, interactions with IκB, and nuclear translocation (34). As shown in Fig. 4E and F, both p65RHD and p50RHD were efficiently immunoprecipitated by US3. These results suggest that US3 interacts with the RHD domains of p65 and p50, phosphorylating the above transcriptional activators and blocking their nuclear translocations, thereby leading to the inhibition of the DNA sensing signaling.

### MDV US3 phosphorylates p65 and p50 and reduces their nuclear translocation

**Fig 5:**
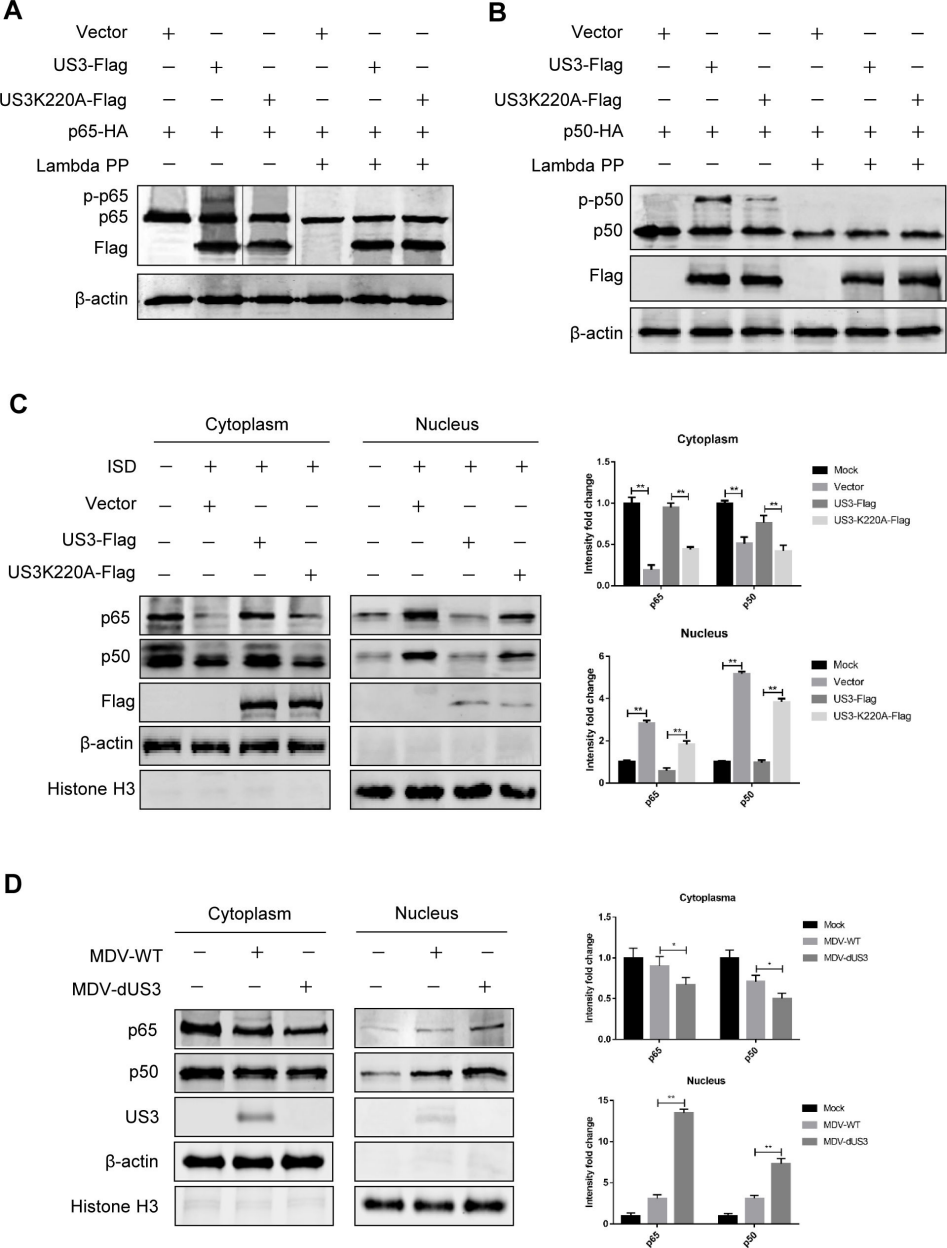
MDV US3 phosphorylates p65 and p50 and reduces their nuclear translocation. (**A, B**) The p65-HA (**A**) or p50-HA (**B**) expression plasmid was co-transfected with an empty vector, US3- or US3K220A-expressing plasmid to DF-1 cells. Forty-eight hours later, cells were lysed and left untreated or treated with Lambda protein phosphatase (Lambda PP) for further Western blot analyses with indicated antibodies. (**C**) DF-1 cells were transfected and treated with ISD, and the cell lysates were separated into cytoplasmic and nuclear extracts. The p65 and p50 protein levels in the cytoplasm and nucleus were analyzed by Western blot. (**D**) CEFs were infected with wild-type MDV (MDV-WT) or the US3-deficient MDV (MDV-dUS3) for 24 h. The cell lysates were separated into cytoplasmic and nuclear extracts and analyzed with immunoblot assays with the indicated antibodies. The data represent results from one of the triplicate experiments. *P < 0.05, **P < 0.01.

Subsequently, we investigated whether p65 and p50 were MDV US3 protein kinase substrates. DF-1 cells were transfected with p65 or p50 expression plasmid alone or with US3- or US3K220A-encoding plasmid to test this hypothesis. As shown in Fig. 5A, a slower-migrating form of p65 was observed under the expression of US3 but not US3K220A, and the slowly migrating p65 was eliminated by Lambda PP treatment. Similarly, US3 expression also induced a slower-migrating form of p50, which was significantly reduced under US3K220A expression and eliminated following Lambda PP treatment (Fig. 5B). These results indicated that p65 and p50 were phosphorylated by US3 and the kinase activity of US3 was indispensable.

The nuclear translocation of p65 and p50 is crucial for the transcription of NF-κB-related genes. To determine if US3 abrogates the trafficking of these NF-κB subunits, DF-1 cells were transfected with US3- or US3K220A-encoding plasmid, and the ISD-stimulated nuclear trafficking of endogenous p65 and p50 was monitored. As shown in Fig. 5C, stimulation with ISD led to increased levels of p65 and p50 in the nuclei. However, ectopic expression of US3 reduced nuclear trafficking of p65 and p50 induced by ISD, and the US3 kinase-dead mutation abolished the inhibition effects of US3 on p65 and p50 nuclear translocation. To investigate the above functions of US3 in the context of MDV infection, the US3-deficient MDV was inoculated in chicken embryo fibroblasts (CEFs). Comparing with the cells infected with wild-type MDV, higher levels of p65 and p50 were detected in the nuclei after infection with MDV-dUS3 (Fig. 5D). These results suggest that MDV US3 induces the phosphorylation of p65 and p50 and reduces their nuclear translocation.

### US3 contributes to the evasion of innate immune response to MDV

**Fig 6:**
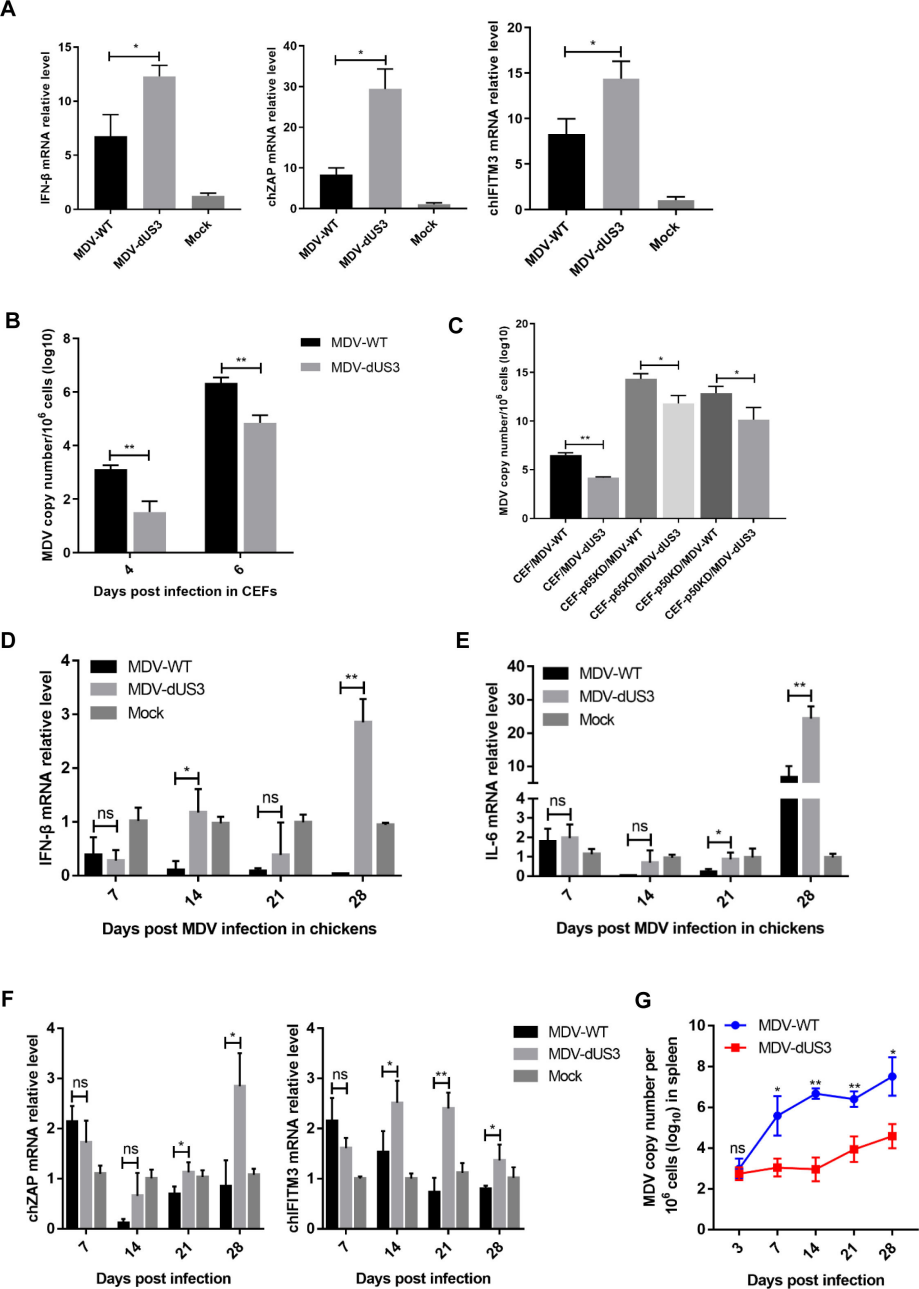
US3 deficiency facilitated IFN-β induction and attenuated MDV replication in chickens. (A) CEFs were infected with wild-type MDV (MDV-WT) or the US3-deficient MDV (MDV-dUS3) for 12 h before the analysis of IFN-β, ZAP, and IFITM3 mRNA levels. (B) The growth properties of MDV-WT and MDV-dUS3 in CEF cell cultures. (C) The indicated cells were infected with wild-type MDV or MDV-dUS3, respectively, and the MDV viral titers were tested with qPCR after 6 days of infection. (D–F) Chickens were infected with MDV-WT and MDV-dUS3, and IFN-β (D), IL-6 (E), ZAP, and IFITM3 (F) production in chickens were measured by qPCR at the indicated time points during MDV infection. (G) Chickens were infected with MDV-WT and MDV-dUS3, and virus genome copy numbers in the spleen were monitored at the indicated time points during MDV infection. ns, no significant difference, *P < 0.05, **P < 0.01, ***P < 0.001.

We examined the expression of IFN-β and the IFN-stimulated genes in cells infected with the wild-type or US3-deficient MDV. The results showed that the mRNA levels of IFN-β and the IFN-stimulated genes ZAP and IFN-inducible transmembrane protein 3 (IFITM3) induced by MDV-dUS3 were notably higher than those induced by wild-type MDV in CEFs (Fig. 6A). Further studies revealed that the replication ability of MDV-dUS3 was decreased compared to that of wild-type MDV in CEFs (Fig. 6B). We further transfected CEFs with a p65-specific small interfering RNA (siRNA) or a p50-specific siRNA, and the replication of MDV-WT and MDV-dUS3 was compared with that in cells transfected with a nontargeting siRNA. As shown in Fig. 6C, MDV-dUS3 produced from wild-type CEFs decreased approximately 1.56-fold in comparison to wild-type MDV, whereas MDV-dUS3 produced from p65-knockdown cells (CEF-p65KD) or p50-knockdown cells (CEF-p50KD) decreased approximately 1.21-fold or 1.26-fold in comparison to wild-type MDV. These results suggested that US3 promotes MDV replication in a NF-κB-dependent pathway. Next, specific-pathogen-free (SPF) chickens were infected with wild-type MDV or the US3-deficient MDV, and then the expression of IFN-β, the IFN-stimulated genes, and the NF-κB-regulated cytokine IL-6 was detected. Compared with wild-type MDV, the US3-deficient MDV induced significantly higher levels of IFN-β in chickens after 14 and 28 days of MDV infection (Fig. 6D). The IL-6, ZAP, and IFITM3 expression levels induced by MDV-dUS3 were also remarkably higher than that induced by wild-type virus infection (Fig. 6E and F).

Furthermore, the viral DNA loads in the infected chickens were analyzed by qPCR. As shown in Fig. 6G, the MDV-dUS3 virus replicated at the parental MDV-WT level during early cytolytic infection at 3 days post-infection (dpi). However, the MDV-dUS3 viral loads measured beyond 7 dpi were reduced by 100- to 1,000-fold compared with MDV-WT. These results indicated that US3 is dispensable for early cytolytic infection; nonetheless, it might play a role in the later phases of MDV infection, including latency, reactivation, and transformation. These results suggest that the deletion of MDV US3 attenuates the replication of MDV in chickens. Altogether, these studies demonstrate that US3 interacts with p65 and p50, abrogating IFN-β production and evading antiviral innate immunity during MDV infection.

## DISCUSSION

The innate immune response is pivotal for host defense against viral infection. During herpesviruses infection, viral DNA is released into the cytoplasm, which triggers the activation of the cGAS-STING DNA sensing pathway, resulting in the activation of IRF3 and NF-κB (9, 10). IFNs and inflammatory chemokines are then produced to suppress the replication of herpesviruses. Viruses have developed multiple immune evasion strategies to inhibit IFN-I production and facilitate viral infection and replication (35, 36). The strategies were especially important for MDV because periodic lytic replication and *de novo* infection occur naturally and are required for long-term persistence and pathogenesis in its hosts. Compared to mammalian viruses, strategies used by chicken DNA viruses to block the cGAS-STING pathway were not well understood. We recently reported that the major oncoprotein of MDV, Meq, inhibited this pathway through interaction with STING and IRF7, preventing the associations of STING-TBK1 and STING-IRF7 and suppressing IRF7 activation and IFN-I production (37). The VP23 and RLORF4 proteins encoded by MDV abrogated the DNA Sensing Pathway by antagonizing IRF7 or NF-κB activation (32, 38). In the present study, based on a screen of MDV ORFs, we identified the MDV protein kinase US3 as an efficient inhibitor of IFN-I induction. By interacting with the NF-κB subunits p65 and p50, US3 suppresses the nuclear translocation of these transcription factors, which eventually inhibits IFN-I production during MDV infection.

Previous reports indicated that alphaherpesvirus-encoded US3 protein kinase is a multifunctional protein essential for virus infection and replication (23). Several cellular and viral proteins have been identified as HSV-1 US3 substrates, including the viral proteins UL31, UL34, gB, and dUTPase (39–41), and cellular proteins, such as IRF3, p65, histone deacetylase 1 (HDAC-1), and HDAC-2, β-catenin, and the type II kinesin motor protein KIF3A (42–44). MDV US3 was first described in 1993 and was shown to share the functions of other alphaherpesviruses-encoded US3 orthologs in regulating virion morphogenesis, apoptosis, and host cytoskeleton structure (45–47). It has been reported that MDV US3 interacts with and phosphorylates viral oncoprotein Meq and pp38 (33, 47). Cellular proteins cAMP response element-binding protein (CREB) and HDAC1 and 2 are also substrates of MDV US3 (33, 48). In addition, MDV US3 was recently found to disrupt the promyelocytic leukemia protein nuclear bodies in a US3 kinase activity-dependent manner (49). A previous study reported that MDV US3 facilitates viral replication by targeting IRF7 to block IFN-β production (50). In the present study, MDV US3 was further shown to act as an efficient inhibitor of the chicken DNA sensing pathway by interacting with the essential transcription factors p65 and p50. Our results indicated that the evasion of host antiviral innate immunity by US3 is important for MDV replication in chickens.

Phosphorylation plays an important role in regulating protein functions and affects various viral and cellular processes, including gene regulation, protein stability, and protein interactions (51). Alphaherpesvirus US3 can phosphorylate different target proteins, and the kinase activity is critical for its functions in controlling multiple cellular and viral replication processes. For example, the kinase activity of HSV-1 US3 is important for its shuttling between the nucleus and cytoplasm (23). The function of MDV US3 to protect cells from apoptosis is dependent on its kinase activity (47, 52). Recent studies also reported that MDV US3 kinase activity is important for expressing several MDV genes and its interaction with MDV oncoprotein Meq (33). In this study, our results suggest that the kinase activity of MDV US3 is essential for its function of inhibiting IFN-β and IL-6 production. The kinase-dead US3 mutant US3K220A failed to hyperphosphorylate p65 and p50, or prevent the nuclear localization of NF-κB, indicating that the kinase-dead mutant abolished the inhibitory activity of MDV US3 on the DNA sensing signaling.

The transcription of IFN-I depends on both IRFs and NF-κB that bind to distinct regulatory domains in the promoter. NF-κB is also critical for inducing the production of various inflammatory chemokines and interleukins. Thus, viruses are more likely to evolve strategies to counteract the activation of NF-κB. Previous studies demonstrated that the viral proteins, such as HSV-1 UL24, UL36USP, and ICP0, abrogated NF-κB activation (53–55). We previously reported that MDV RLORF4 antagonized NF-κB activation, thus inhibiting DNA-sensing signaling in chicken cells (38). Here, we demonstrated that MDV US3 abrogates NF-κB activation in a kinase activity-dependent manner, further expanding the functions of US3 and our knowledge about the mechanisms of MDV innate immunity evasion during infection in chickens. It is worth noting that the transcription factors downstream in the DNA sensing pathway, such as p65 and p50, are shared by the IFN-I signal pathways mediated by other PRRs, including Toll-like receptors (TLRs) recognizing endosomal nucleic acids and the retinoic acid-inducible gene I-like receptors (RLRs) recognizing viral RNA. Therefore, it is reasonable to consider that viral proteins targeting these transcription factors in the DNA sensing pathway may also affect TLRs- and RLRs-mediated signals through a similar mechanism, leading to the inhibition of IFN-I production triggered by bacteria and RNA virus infection. We inferred that MDV US3 functioned cooperatively with other viral proteins to inhibit host antiviral innate immunity, which could be one of the reasons why birds infected with MDV exhibit immunosuppression and are more susceptible to concurrent or secondary viral or bacterial infections.

In conclusion, we describe a new function of MDV protein kinase US3 that inhibits the DNA sensing signaling pathway. Our results reveal that MDV US3 efficiently inhibited DNA sensor-mediated IFN-β and IL-6 production due to the inhibition of NF-κB activation by interacting with and phosphorylating the NF-κB subunits p65 and p50. These findings will help our understanding of the interaction between MDV replication and the DNA-sensing signal pathway and provide insights into how avian herpesviral kinase counteracts host antiviral innate immunity to ensure viral replication and spread.

## MATERIALS AND METHODS

### Animals and ethics statement

The SPF chicken eggs and duck eggs were purchased from National Laboratory Poultry Animal Resource Center (Harbin, China). Ten-day-old SPF chicken and duck embryos were used to prepare primary CEFs and duck embryo fibroblasts (DEFs). According to the Guide for the Care and Use of Laboratory Animals of the Ministry of Science and Technology of China, this study was carried out. The use of SPF eggs, embryos, and chickens and the animal experiments were approved by the Animal Ethics Committee of Harbin Veterinary Research Institute of the Chinese Academy of Agricultural Sciences and performed following animal ethics guidelines and approved protocols [SYXK (Hei) 2017-009].

### Cells, viruses, and antibodies

DF-1 (ATCC CRL-12203) and HEK293T (ATCC CRL-3216) cells were cultured in Dulbecco’s Modified Eagle’s Medium (DMEM, Gibco, C11995500BT) containing 10% fetal bovine serum (FBS, Sigma-Aldrich, F8318). Primary CEFs and DEFs were prepared from 10-day-old SPF embryos and cultured in DMEM supplemented with 5% FBS. The virulent MDV GA strain (GenBank no. AF147806) and HVT FC126 strain (GenBank no. AF291866) were propagated in CEFs prior to this study. Commercially available antibodies include mouse anti-Flag (Sigma-Aldrich, F1804), rabbit anti-HA (Sigma-Aldrich, H6908), mouse anti-c-Myc (Sigma-Aldrich, M4439), rabbit anti-c-Myc (Sigma-Aldrich, C3956), mouse anti-actin (Sigma-Aldrich, A1978), rabbit anti-Histone H3 (Abcam, ab201456), rabbit anti-p65 (Abcam, ab32536), and rabbit anti-p50 (Abcam, ab283688). The rabbit anti-US3 antibodies were prepared in our laboratory. ISD was purchased from InvivoGen (San Diego, tlrl-isdn).

### Plasmid construction

The MDV US3 gene was amplified from the genome of the virulent MDV GA strain and cloned into the pCAGGS vector for expression. Plasmids encoding chicken cGAS, STING, IKKα, IKKβ, p65, and p50 were constructed as previously described (32, 38). The chicken IFN-β promoter-luciferase reporter IFN-β-Luc was constructed by inserting the −158 to +14 fragment of the chicken IFN-β promoter into the pGL3-basic vector (56). The pNF-κB-Luc reporter contained four copies of the NF-κB-binding positive regulatory domain (GGG AAT TCT C).

### Transfection and dual-luciferase reporter assays

To determine chicken IFN-β promoter and NF-κB binding activities, DF-1 cells were co-transfected with a firefly luciferase reporter plasmid (IFN-β-Luc or NF-κB-Luc) and Renilla luciferase reporter pRL-TK, which served as an internal control, with or without expression plasmids, as indicated, using the TransIT-X2 dynamic delivery system (Mirus, Madison, WI, USA). At 36 h posttransfection, cells were lysed, and samples were assayed for firefly and Renilla luciferase activity using the dual-luciferase reporter assay system (Promega, Madison, WI, USA). Relative luciferase activity was normalized to Renilla luciferase activity. The reporter assays were repeated at least three times.

### Real-time qPCR

Total RNA was extracted using RNAiso Plus reagent (TaKaRa, Otsu, Japan). Reverse transcription was performed using ReverTra Ace qPCR RT Kit (Toyobo, Osaka, Japan). The quantity of each cDNA was determined by real-time qPCR using Thunderbird SYBR qPCR mix (Lucigen, Madison, WI, USA) and analyzed with the LightCycler 480 system (Roche, Basel, Switzerland). Specific primers for IFN-β, IL-6, chicken ZAP, and IFITM3 were synthesized by Invitrogen (Shanghai, China), and the relative mRNA levels of these genes were normalized to the chicken β-actin mRNA level in each sample. The fold differences between the treated samples and mock samples were calculated. To determine MDV viral titers, total DNA was extracted using the AxyPrep BodyFluid Viral DNA/RNA Miniprep Kit (Corning Life Sciences, Shanghai, China) and tested with real-time qPCR by measuring the copy numbers of the MDV Meq gene as an MDV genome target and the chicken ovotransferrin gene as a reference, as described previously (57). All controls and treated samples were examined in triplicate on the same plate.

### ELISA

The IFN-β and IL-6 protein levels in cell cultures were analyzed using chicken IFN-β or IL-6 ELISA kit (USCN Life Science, Wuhan, China) according to the manufacturer’s instructions.

### Coimmunoprecipitation and Western blot assays

The expression plasmids with Flag, HA, or Myc tags were transfected into HEK293T or DF-1 cells using the TransIT-X2 dynamic delivery system. At 36 h posttransfection, cells were lysed in ice-cold Pierce IP buffer containing protease inhibitor cocktail (Thermo Fisher Scientific, Waltham, MA, USA). The lysates were obtained by centrifugation and incubated overnight with the indicated antibodies at 4°C. Protein G Sepharose beads (Roche) were added, and samples were incubated for another 6 h. The beads were washed six times with phosphate-buffered saline and boiled in sodium dodecyl sulfate-loading buffer before analysis by Western blot with the indicated antibodies.

For Western blot, whole-cell lysates were obtained by lysing cells in NP-40 lysis buffer (Beyotime, Beijing, China). The cytoplasmic and nuclear proteins were extracted using NE-PER nuclear and cytoplasmic extraction reagents (Thermo Fisher Scientific). Protein concentrations were determined with a bicinchoninic acid protein assay kit (Thermo Fisher Scientific). The proteins were separated by electrophoresis on 12% SDS-polyacrylamide gels, transferred onto nitrocellulose membranes, and incubated with the indicated primary and secondary antibodies. Images were acquired with the Odyssey infrared imaging system (LI-COR Biosciences, Lincoln, NE, USA).

### *In vitro* kinase and dephosphorylation assays

To detect US3 kinase activity, the cells transfected with US3-expression plasmid and the plasmids encoding p65, or p50, or infected with MDV-WT or MDV-dUS3 were lysed. The supernatants were obtained and subjected to a phosphate gel for immunoblot with anti-p65 or anti-p50 antibodies. Phosphate gel was 10% acrylamide gel with the addition of 5 mM phosphate-binding tag (Phos-tag, ApexBio Technology, USA) and 10 mM Mn^2+^ in the resolving gel. Before transferring, the gel was washed three times every 10 min in the transfer buffer containing 10 mM EDTA, which is required to increase the transfer efficiency. Phos-tag and Mn^2+^ cooperate to bind a phosphorylated protein; when the phosphorylation levels are increased, the migration velocity of this protein is slower. Therefore, non-phosphorylated and phosphorylated proteins could be separated in the gel.

To confirm that MDV US3 mediates the phosphorylation of p65, and p50, cell lysates were subjected to a dephosphorylation assay using the Lambda Protein Phosphatase (Lambda PP, New England Biolabs) as described previously (46). The samples with or without Lambda PP treatment were then analyzed by SDS-PAGE and Western blot assays.

### Generation of US3-deficient MDV

Taking advantage of a previously established fosmid-based rescue system for the virulent MDV GA strain (34), a US3-deficient MDV was constructed. Six fosmid clones, GA1 to GA6, containing sequences encompassing the entire genome of GA, were included in this system. Fosmid GA6, containing the coding sequence of US3, was used to delete the US3-encoding sequence with the Counter-Selection BAC Modification Kit (Gene Bridges, Heidelberg, Germany). The US3-deficient fosmid clone, designated GA6-dUS3, was co-transfected with the other five parental fosmid clones into primary DEFs using the calcium phosphate procedure. Five days after transfection, cells were trypsinized, seeded on DEFs, and monitored for cytopathic effects. Viral stocks were subsequently generated in DEFs for further analysis. The rescued virus was verified by PCR and sequencing, and the deficient expression of US3 was analyzed by Western blot with anti-US3 antibodies.

### RNA interference

siRNAs specifically targeting chicken p65 and p50 as well as a scrambled negative control siRNA were synthesized by GenePharma (Shanghai, China). The siRNA transfections were performed in CEFs using the TransIT-X2 dynamic delivery system (Mirus, Madison, WI, USA) according to the manufacturer’s instructions. After 12 h of transfection, cells were infected with the wild-type MDV or MDV-dUS3 for further analysis.

### Animal experiments

To determine the effects of MDV infection on the production of IFN-β, IL-6, and downstream ISGs, each group of 25 one-day-old SPF chickens was inoculated subcutaneously with 2,000 PFUs of MDV-WT or MDV-dUS3, and a third group injected with DMEM was included as a mock control. Five birds from each group were humanely euthanized on days 3, 7, 14, 21, and 28, and spleen samples were collected. Total RNA was extracted to analyze the expression of IFN-β, IL-6, and ISGs, and DNA was extracted to analyze viral DNA copy numbers. For MDV pathogenicity analyses, groups of 20 one-day-old SPF chickens were inoculated with MDV-WT, MDV-dUS3, or DMEM and examined for clinical signs and mortality for 12 weeks. All chickens were necropsied at the time of death or at the end of the experiment to evaluate the presence of MDV-specific lesions in visceral organs and nerves.

### Statistical analysis

All experiments were performed at least three times unless otherwise indicated; data are presented as the means ± standard deviations (SD). Statistical significance between groups was determined by Student’s *t*-test with GraphPad Prism 7.0 software (La Jolla, CA, USA). A *P*-value of <0.05 was considered statistically significant.

## SUPPLEMENTAL MATERIAL

Fig. S1 spectrum.02347-24-s0001.pdf HEK293T cells were transfected with the indicated plasmids for 36 h before coimmunoprecipitation and immunoblot analyses with the indicated antibodies.

ASM does not own the copyrights to Supplemental Material that may be linked to, or accessed through, an article. The authors have granted ASM a non-exclusive, world-wide license to publish the Supplemental Material files. Please contact the corresponding author directly for reuse.
