# Supplementary material for: Marek’s disease virus protein kinase US3 inhibits DNA-sensing antiviral innate immunity via abrogating activation of NF-κB
Source: Microbiol Spectr. 2025 Mar 5;13(4):e02347-24. doi: 10.1128/spectrum.02347-24 (PMC11960123; doi:10.1128/spectrum.02347-24)
Supplement: Fig. S1 — HEK293T cells were transfected with the indicated plasmids for 36 h before coimmunoprecipitation and immunoblot analyses with the indicated antibodies. [file spectrum.02347-24-s0001.pdf]

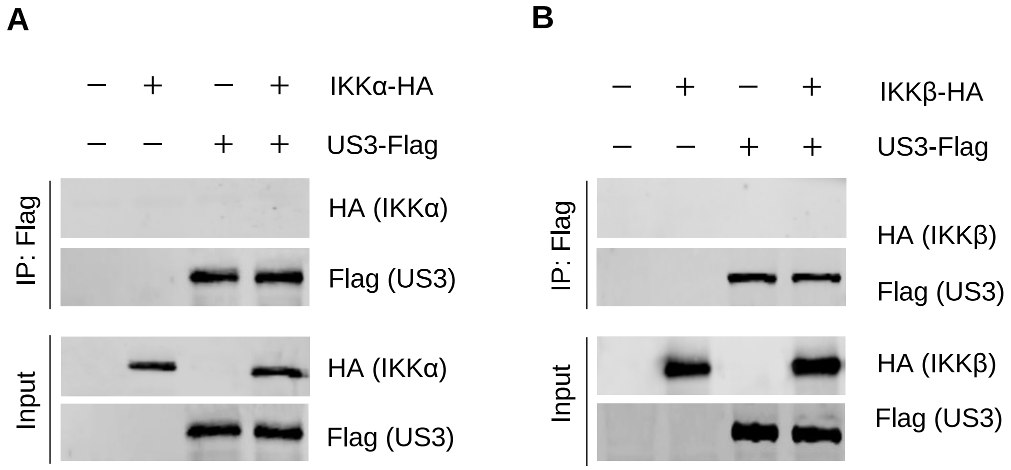

FIG S1 MDV US3 does not interact with IKKα and IKKβ.

HEK293T cells were transfected with the indicated plasmids for 36 h before coimmunoprecipitation and immunoblot analyses with the indicated antibodies.
